# Perturbed Developmental Serotonin Signaling Affects Prefrontal Catecholaminergic Innervation and Cortical Integrity

**DOI:** 10.1007/s12035-018-1105-x

**Published:** 2018-06-09

**Authors:** Lidiane P. Garcia, Josefine S. Witteveen, Anthonieke Middelman, Josephus A. van Hulten, Gerard J. M. Martens, Judith R. Homberg, Sharon M. Kolk

**Affiliations:** 1 Donders Institute for Brain, Cognition, and Behaviour, Centre for Neuroscience, Department of Molecular Animal Physiology, Radboud Institute for Molecular Life Sciences (RIMLS), Radboud University Nijmegen, Geert Grooteplein Zuid 28, 6525 GA Nijmegen, The Netherlands; 2 Donders Institute for Brain, Cognition, and Behaviour, Centre for Neuroscience, Department of Cognitive Neuroscience, Radboud University Nijmegen Medical Centre, Nijmegen, The Netherlands

**Keywords:** Neurodevelopment, Prefrontal cortex (PFC), 5-HT, TH, Cajal-Retzius

## Abstract

Proper development of the medial prefrontal cortex (mPFC), crucial for correct cognitive functioning, requires projections from, among others, the serotonergic (5-HT) and catecholaminergic systems, but it is unclear how these systems influence each other during development. Here, we describe the parallel development of the 5-HT and catecholaminergic prefrontal projection systems in rat and demonstrate a close engagement of both systems in the proximity of Cajal-Retzius cells. We further show that in the absence of the 5-HT transporter (5-HTT), not only the developing 5-HT but also the catecholaminergic system, including their projections towards the mPFC, are affected. In addition, the layer identity of the mPFC neurons and reelin-positive interneuron number and integration are altered in the absence of the 5-HTT. Together, our data demonstrate a functional interplay between the developing mPFC 5-HT and catecholaminergic systems, and call for a holistic approach in studying neurotransmitter systems-specific developmental consequences for adult behavior, to eventually allow the design of better treatment strategies for neuropsychiatric disorders.

**Electronic supplementary material:**

The online version of this article (10.1007/s12035-018-1105-x) contains supplementary material, which is available to authorized users.

## Introduction

Proper functioning of neural systems and correct targeting of their often long projections to distant targets is crucial for cognitive performance. An important distant target of many neurotransmitter systems is the prefrontal cortex (PFC). The PFC is considered critical for executive and higher cognitive functioning [1–3]. Embryonic and early postnatal PFC development is directed by a sequence of intrinsic (e.g., proliferation, migration, and differentiation) and extrinsic (e.g., incoming projections/GABAergic interneurons) events which both can be affected in neurological and psychiatric disorders [1, 4, 5]. During development, the migration of newborn neurons establishes the characteristic inside-out layering of the PFC that furthermore receives numerous projections from various neurotransmitter systems, including the dopaminergic (DA), noradrenergic (NA), and serotonergic (5-hydroxytryptamine or 5-HT) systems [6–10]. It is unclear, however, how these systems interact during development and whether they influence each other. Cognitive and emotional disturbances are often attributed to the perturbed projection to the PFC of more than one neurotransmitter system [11–14], including the catecholaminergic and 5-HT systems, in neurological psychiatric disorders [15–22]. The 5-HT system clearly interacts with the catecholaminergic system in adulthood but it remains to be elucidated how they interrelate during PFC development.

The 5-HT system is one of the earliest to emerge and sends out projections (around E10.5 in mice, E12 in rat) to cortical areas during embryonic development [23, 24]. The rostral raphe comprises 5-HT cell clusters in the dorsal raphe (DR, B6, and B7) and the median raphe (MnR, B5, and B8) that project to the forebrain with predominantly the medial part of the DR projecting to the PFC where they arrive around E16 [25–27]. It has become increasingly clear that 5-HT, but also other neurotransmitters, can act as a neurodevelopmental signal instructing the brain as time proceeds [28–31]. In fact, 5-HT is able to modulate neurodevelopmental processes like proliferation, migration, and differentiation [30, 32]. Within cortical areas, presumptive layer 1 Cajal-Retzius (CR) cells receive serotonergic and noradrenergic synaptic input during embryonic development and might therefore control their functioning [6, 33, 34]. Initially, CR cells secrete reelin which has been proven to be important for the specific inside-out patterning of cortical layers [35–37]. Later, CR cells develop into a heterogeneous population of GABAergic interneurons [38–40]. It remains to be determined however how exactly disturbance of the developing 5-HT system influences the intrinsic neurodevelopmental events of the PFC.

The catecholaminergic system sends out projections to the forebrain approximately at the same time as the 5-HT system (E11.5 in mice, E13 in rat) [9, 41–43]. Tyrosine hydroxylase- or TH-positive axons from the rostral part of the ventral tegmental area (VTA) arrive in the PFC around E15, somewhat earlier than the 5-HT system, in two streams within the subplate (SP) and the marginal zone (MZ) where the CR cells reside [9, 44]. Although it remains speculative to what extent the catecholaminergic projections within the MZ are in synaptic contact with the CR cells, it is known that DA plays a developmental as well as a maturational role in prefrontal areas [45–49]. DAergic projections to the PFC are able to modulate proliferation, migration, and differentiation processes, and any interference during development could contribute to the cortical dysfunction in neuropsychiatric disorders [50].

Until recently, research was focused on understanding the ontogeny and functioning of separate neurotransmitter systems. However, comprehending the development and functioning of the brain in all its facets requires detailed knowledge of how various neural systems interact. In the adult brain, there is a clear interaction between the 5-HT and catecholaminergic projections towards the PFC, especially in the light of their engaged involvement in higher-order cognitive functioning [12, 15, 51]. Anatomically, the two systems considerably overlap in adulthood and seem to receive inputs from one another [52–54]. Less is known, however, about the extent to which the 5-HT and catecholaminergic systems influence each other during neurodevelopment [51, 55].

Here, we describe the parallel development of the 5-HT and catecholaminergic systems in the rat between E16 and P6 with special emphasis on their common projection target, the medial PFC (mPFC). We show that in the absence of the 5-HT transporter (5-HTT), not only the 5-HT but also the catecholaminergic system, including TH-positive projections towards the mPFC, are affected. Within the mPFC, the reelin-containing CR cells are in close proximity to 5-HT and TH-positive fibers, and in the absence of the 5-HTT, they differ in number. We furthermore demonstrate that the identity of especially deep-layer neurons is altered in the 5-HTT^−/−^ rats. Altogether, these data suggest that there is a functional interplay between the 5-HT and catecholaminergic systems during development leading to a distortion of the cytoarchitecture of the PFC. Thus, the possible interplay of multiple neural neurotransmitter systems during development has to be taken into account when studying the etiology of neuropsychiatric disorders.

## Materials and Methods

### Animals

The control neuroanatomical descriptions were performed on wildtype rats of the Wistar background purchased from a commercial breeder (Janvier, Labs, RjHan:WI; Hannover, Germany). The generation of the Slc6a4^1Hubr^ wildtype (5-HTT^+/+^) and mutant rats (5-HTT^−/−^) has been described previously [56]. They were bred onto a Wistar genetic background. The day of the plug was considered to be embryonic day (E)0 and the day of birth to be postnatal day (P)0. All experiments were performed in compliance with the standard ethics guidelines of the European Community and in accordance with the recommendations of the local animal welfare committee (DEC) of the Radboud University. The protocol was approved by the DEC. Male and female embryos and pups were used indiscriminately in all experiments and sacrificed by decapitation.

### Section Preparation and Immunohistochemistry

Brains were rapidly dissected from E16.5, E18.5, and E20.5 embryos, P6 pups, and P25 adolescents, and fixed by immersion for 0.5–1.5 h in 4% paraformaldehyde (PFA) in phosphate-buffered saline (PBS), pH 7.4. After fixation, brains were washed in PBS, cryoprotected in 30% sucrose overnight, frozen in M-1 embedding matrix (Shandon, Thermo Fisher Scientific Inc., Waltham, MA, USA) on dry ice in a plastic cup, and stored at − 80 °C. Cryostat coronal or sagittal sections were cut at 16 μm, mounted as series of 6–8 on Superfrost Plus slides (Thermo Fisher Scientific), air-dried, and stored desiccated at − 20 °C. Cryosections were stained immunohistochemically and imaged as described previously [10, 57] with the following exceptions; incubation of P6 and P25 sections with primary antibodies was done for either 3 h at room temperature (RT) or overnight (ON) at 4 °C. Immunofluorescence was visualized using either an Invitrogen/Thermo Fisher Scientific EVOSTM FL Auto Imaging System with a high-sensitivity CMOS camera or EVOS FL Auto Software or using a Leica DMRA fluorescence microscope coupled with a DFC340FX digital camera and LASAF software. The primary antibodies, dilutions used, and antibody suppliers can be found in Table 1.The nomenclature to describe neurons and axons within different brain areas is as described previously by Schambra et al. [58] and Jacobowitz and Abott [59] and extended as outlined in [9, 10] and in Supplemental Figure 1.

**Table 1:** The primary antibodies, dilutions used, and antibody suppliers

Antibody	Dilution	Company
Rabbit anti-5-hydroxytryptamine (5-HT)	1:1000	Sigma-Aldrich
Mouse anti-Satb2	1:500	Abcam
Mouse anti-reelin	1:500	Chemicon
Mouse anti-Cux1	1:300	Abcam
Rabbit anti-tyrosine hydroxylase (TH)	1:1000	Millipore
Chicken anti-tyrosine hydroxylase (TH)	1:500	Abcam
Rabbit anti-Tbr1	1:500	Abcam

### Data Analysis

All data analyses were performed in a blinded fashion without knowledge of the animal’s genotype. For assessing 5-HT- or TH-positive axon length and number of layer marker-positive neurons within the various subareas of the medial PFC (mPFC) of *5-HTT*^*+/+*^ and of *5-HTT*^−/−^ rats, three to five P6 pups were analyzed and two to four well-spaced (120 μm) sections at the same neuroanatomical level were imaged. A 0.1-mm-wide rectangle spanning the prefrontal wall was placed over the center of the subarea (either infralimbic, IL; prelimbic, PL; or cingulate cortex, Cg) of the mPFC. The overall cortical length of a subarea was divided into ten equal bins [bin 1 within the deep cortical layers and bin 10 within the presumptive layer I] within this rectangle, and 5-HT- or TH-positive axon length or number of layer marker-positive neurons were measured within each bin using ImageJ software including the NeuronJ plugin (NIH, Bethesda, USA). Data were normalized to a total length per bin, or to a percentage of the total number of cells and averaged for each pup. To better visualize and compare 5-HT and/or catecholaminergic innervation of wildtype and mutant mPFC, reconstructions of the individual fibers from two to three consecutive sections were obtained bilaterally using the NeuronJ plugin. Data were statistically analyzed by one-way ANOVA (*α* = 5%) using Graphpad Prism 6/Excel data analysis toolkit and expressed as means ± SEM.

Surface area of TH^+^ (tyrosine hydroxylase, rate-limiting enzyme responsible for DA synthesis) area within the ventral midbrain was measured by dividing the area along the midline. The surface area was then measured in μm^2^ with Image J and averaged between left and right of two to three well-spaced sections. For each *n*, the same neuroanatomical level was chosen in terms of rostral-to-caudal extent. The measurements of 5-HT^+^ surface area of the B7 and B8 nuclei in the hindbrain was performed in a similar fashion with the exception that the entire nucleus was measured and not divided along the midline. 5-HT^+^ cell numbers of the B7 and B8 nuclei were counted in Adobe Photoshop in the same area used to measure the surface area in two to four well-spaced sections. Again, for each *n*, the same neuroanatomical level was chosen in terms of rostral-to-caudal extent. Data were statistically analyzed by one-way ANOVA (*α* = 5%) using Graphpad Prism 6/Excel data analysis toolkit and expressed as means ± SEM.

## Results

### Developing 5-HT and Catecholaminergic Systems Targeting the mPFC

In rats, 5-HT neurons start extending axons by E12 and TH^+^ axons by E13 [9, 23]. Both neurotransmitter systems send out ascending axonal projections to distant forebrain targets including the mPFC [9, 23, 43]. The ontogeny of both systems has been described in detail for each system individually, however little is known of the concurrent development of both systems.

**Fig. 1:**
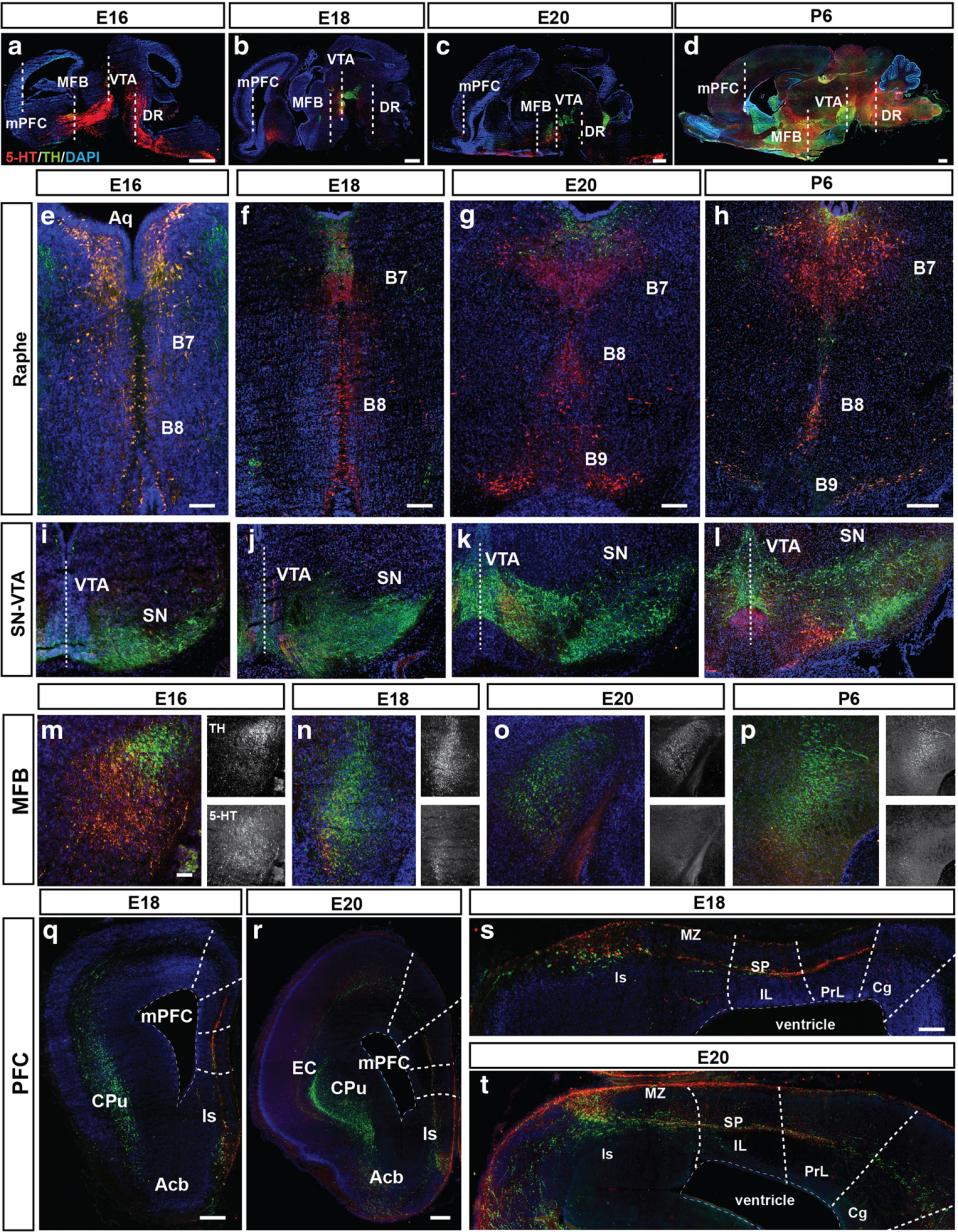
Developing 5-HT and catecholaminergic systems target the mPFC. Sagittal cryosections of E16 (**a**), E18 (**b**), E20 (**c**), and P6 (**d**) rat brains immunostained for 5-HT (red) and TH (green) and stained with DAPI (blue) to visualize cell nuclei showing the 5-HT and the catecholaminergic developing systems. Dotted lines indicate the coronal section levels. Enlargements of coronal cryosections of E16 (**e**), E18 (**f**), E20 (**g**), and P6 (**h**) rat brains immunostained for 5-HT (red) and TH (green) and stained with DAPI (blue) showing the DR with the B7, B8, and/or B9 5-HT-positive cell clusters closely intermingled with TH-positive neurons and fibers. Enlargements of coronal cryosections of E16 (**i**), E18 (**j**), E20 (**k**), and P6 (**l**) rat brains immunostained for 5-HT (red) and TH (green) and stained with DAPI (blue) showing the rostral ventral tegmental area (rVTA) and the substantia nigra (SN) with the TH-positive cell clusters/fibers closely intermingled with 5-HT-positive neurons and fibers. Dotted lines indicate the midline. Enlargements of coronal cryosections of E16 (**m**), E18 (**n**), E20 (**o**), and P6 (**p**) rat brain immunostained for 5-HT (red) and TH (green) and stained with DAPI (blue) showing the medial forebrain bundle (MFB) with TH residing mainly in dorsal fascicles while 5-HT is present mostly within more caudal ones. Coronal (half shown) cryosections of E18 (**q**) and E20 (**r**) rat brains immunostained for 5-HT (red) and TH (green) and stained with DAPI (blue) showing the forebrain targets of both systems including the caudate putamen (CPu), the lateral septum (ls), the nucleus accumbens (Acb), and the medial prefrontal cortex (mPFC). Enlargements of the mPFC region of E18 (**s**) and E20 (**t**) rat brains showing a TH/5-HT-positive stream above the subplate (SP) and one in the marginal zone (MZ) in all three prefrontal subdomains (infralimbic, IL; prelimbic, PL; and cingulate, Cg). ls, lateral septum. Bar in **a**–**d**, 500 μm; **e**, 200 μm; **f**, 300 μm; **g**, 250 μm; **h**, 200 μm; **i**–**l**, 300 μm; **m**–**p**, 200 μm; **q** and **r**, 300 μm; **s** and **t**, 250 μm

In order to examine the relationship between the developing 5-HT and catecholaminergic system, we immunostained cryosections of developing rat brains (E16, E18, E20, and P6) for TH and 5-HT. Sagittal sections suggest a close interrelationship of especially ascending TH^+^ and 5-HT^+^ axons (Fig. 1a–d). When we took a closer look at the origin of both ascending neural systems, the DA midbrain and the 5-HT DR and MnR at the coronal level, we were able to observe a close contiguity between the TH^+^ and 5-HT^+^ axons (Fig. 1e–t). Within the developing DR and MnR, besides the 5-HT neuron clusters, a combination of both TH^+^ axons as well as TH^+^ cell bodies could be observed (Fig. 1e–h). At E16, we observed overlapping positioning of the majority of the TH- and 5-HT-positive neurons (Fig. 1e). At E18, most of the TH^+^ neurons and fibers could be observed dorsally within or near the B7 of the DR (Fig. 1e–h). Within the developing DA midbrain at E16, 5-HT axonal projections were closely intermingled with the developing and still migrating TH^+^ neurons in both the substantia nigra (SN) and the VTA. The latter seemed to be more innervated by 5-HT projections throughout the course of development (Fig. 1i–l).

TH^+^ and 5-HT axons travel together towards forebrain targets and they run in parallel within the median forebrain bundle (MFB). We observed that TH^+^ axons bundle in larger fascicles and reside more dorsal within the extent of the MFB when compared to the 5-HT axons (Fig. 1m–p). The 5-HT axons are located more ventral within the MFB and seemed to get more varicose as development proceeds (Fig. 1m–p). However, a large proportion of the TH^+^ and 5-HT axons coincide, suggesting a close contiguity within the MFB during development (Fig. 1m–p).

Eventually, both neural systems reached numerous forebrain targets including the mPFC (Fig. 1q–t). Especially within the region of the lateral septum (ls), the catecholaminergic system concurred with the 5-HT system although there was only partial overlap. Within the mPFC, there are two main fascicle paths of TH^+^ and 5-HT axon bundles. At E18, the first path was observed as a robust bundle of fascicles and individual 5-HT-positive fibers were detected above the subplate (SP) of all three mPFC subdomains (infralimbic, IL; prelimbic, PL; and Cingulate, Cg), although the fibers were less prominent within SP of the Cg (Fig. 1q–t). TH-positive fibers could also be observed at E18 but were less conspicuous and fasciculated (Fig. 1q–t). The other path of concurring TH^+^ and 5-HT^+^ fibers was found within the marginal zone (MZ) or the presumptive layer 1 where again the presence of 5-HT fibers exceeded that of the TH^+^ ones (Fig. 1q–t). At E20, the innervation within the mPFC subdomains had increased in both paths and both systems innervated the cortical plate (CP) (Fig. 1s, t).

In summary, there is a close intercalation and proximity of the catecholaminergic system and 5-HT system during development. They both innervate the mPFC in a similar pattern and time frame. Although the catecholaminergic system arrives earlier, the 5-HT system remains more prominent throughout development.

### Close Proximity of DA and 5-HT Projections Within the Developing mPFC

There is a vast amount of information on the innervation of the mPFC by each of the catecholaminergic and 5-HT systems. However, less is known about the coinciding innervation of the mPFC during development.

To address the coinciding localization and possible interaction of the catecholaminergic and the 5-HT system within the developing mPFC, we focused on the immunoreactive TH and 5-HT fibers within the different aspects of the mPFC subdomains. To show the proximity and intercalation of both neural systems, camera lucida drawings were obtained. At E16, no TH^+^ or 5-HT^+^ fibers could be observed within all cortical aspects of the individual subdomains (data not shown). At E18, the two above-mentioned paths could be observed especially in the IL and PL (shown) with TH^+^ and 5-HT^+^ varicose fibers running above the SP and within presumptive layer 1 (Fig. 2b, e, and h). Hardly, any innervation of the CP was present at this time point. At E20, both streams were still prominent with 5-HT^+^ exceeding TH^+^ fibers in the presumptive layer 1 and to a lesser extent above the SP (Fig. 2c, f, i, and k). Substantial innervation of the CP of mostly 5-HT^+^ fibers could also be observed. At P6, extensive innervation by both neurotransmitter systems of all cortical aspects could be observed (Fig. 2d, g, j, and k). Yet, there was a clear 5-HT- and TH-positive band within layer I, which is most likely in close proximity of CR cells (Fig. 2b–j). TH^+^ and 5-HT^+^ axons seemed to run in close proximity especially early within development but, as was also clear from the camera lucida drawings, they appear to have their own distinct target cells across layers.

**Figure:**
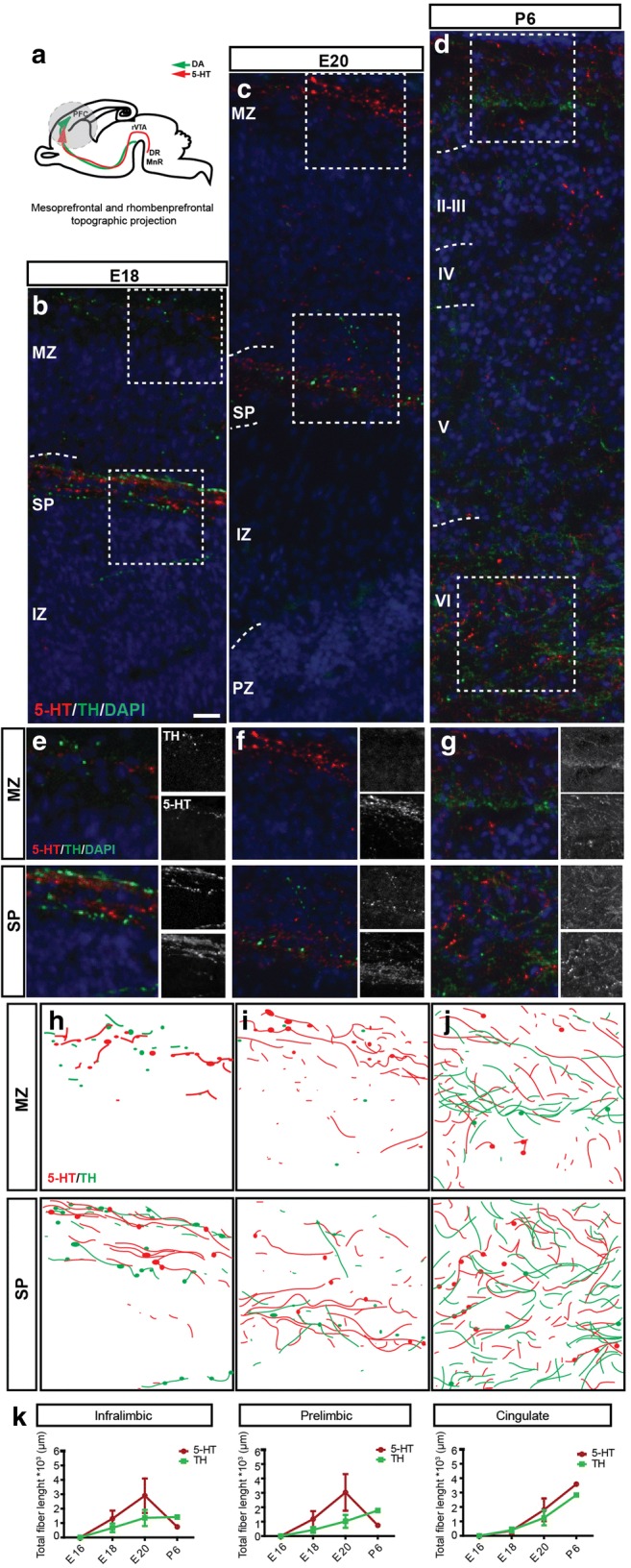

Thus, within the developing mPFC, both the catecholaminergic and the 5-HT systems are present and innervate cortical areas via the SP and MZ. Later, a large variety of neurons within the mPFC cortical layers get innervated by both the catecholaminergic as well as the 5-HT system.

### The Developing Catecholaminergic System is Affected in the Absence of the 5-HTT

**Fig. 3:**
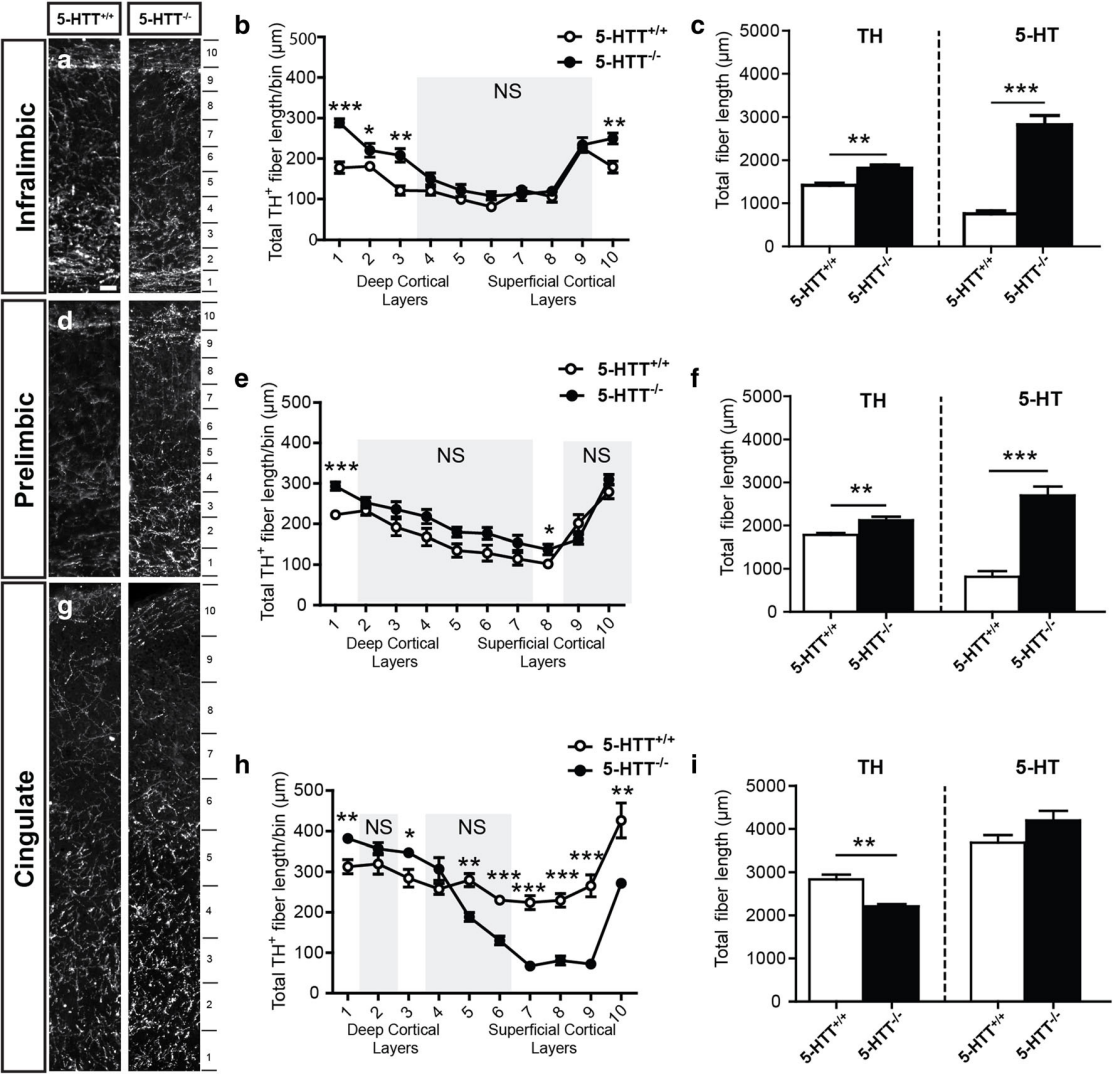
Catecholaminergic innervation of the mPFC affected in 5-HTT-deficient rat model. Enlargements of cryosections of P6 5-HTT^+/+^ and 5-HTT^−/−^ rat brains showing prefrontal swatches of the IL (**a**), PL (**d**), and the Cg (**g**) immunostained for TH (white). Quantification of the TH^+^ fiber length (in μm) within the bins indicated in **a**, **d**, and **g** in the IL (**b**), PL (**e**), and Cg (**h**) of 5-HTT^−/−^ compared to 5-HTT^+/+^ pups confirming the qualitative observations. The gray boxes represent the non-significant (NS) bins. Graphs in **b**, **e**, and **h** show average total length of TH-positive fibers per bin ± SEM. One-way ANOVA, ^*^p < 0.05, ^**^p < 0.01, ^***^p < 0.001. Quantification of the total TH-positive (left) as compared to the total 5-HT-positive fiber length (in μm) over the complete length of the prefrontal swatch in the IL (**c**), PL (**f**), and Cg (**i**) of 5-HTT^−/−^ compared to 5-HTT^+/+^ pups. Graphs in **c**, **f**, and **i** show average total length of TH- and 5-HT-positive fibers ± SEM. One-way ANOVA, ^**^p < 0.01, ^***^p < 0.001. Bar in all, 50 μm

It is well accepted that 5-HT has an important neurodevelopmental role [30, 60–62]. In previous work, we demonstrated that in the absence of 5-HTT, the amount of 5-HT-positive fibers increased dramatically within certain cortical layers of the IL and PL and to a lesser extent of the Cg ([10] and Fig. 3, Supplemental Figure 1). This raises the question of how elevated levels of extracellular 5-HT during development might influence other neural systems.

To address whether the catecholaminergic system would be affected, we studied P6 brains of the 5-HTT mutant rat model. First, we tried to recapitulate our previous results: the 5-HT innervation of the subdomains of the mPFC was higher in 5-HTT^−/−^ animals (*n* = 5) compared to wildtype (*n* = 5). Indeed, we again observed a significant increase of total 5-HT innervation in both the IL (*p* = 0.000016) and PL (*p* = 0.000059) and to a lesser extent in the Cg (*p* = 0.11049; Fig. 3c, f, i). Strikingly, we discovered that the catecholaminergic innervation of the mPFC subdomains is also affected in 5-HTT^−/−^ animals. Within the IL and PL, we found a significant increase of TH^+^ innervation in especially the deeper layers and in bins 10 and 8 (more superficial layers) for the IL and PL of 5-HTT^−/−^ animals, respectively (Fig. 3a, b, d, and e). Interestingly, the TH^+^ innervation of the Cg tended to be higher in the deeper cortical layers whereas it was significantly lower in the more superficial layers, mimicking the results we obtained before for the 5-HT innervation [10] (Fig. 3g–h).

**Fig. 4:**
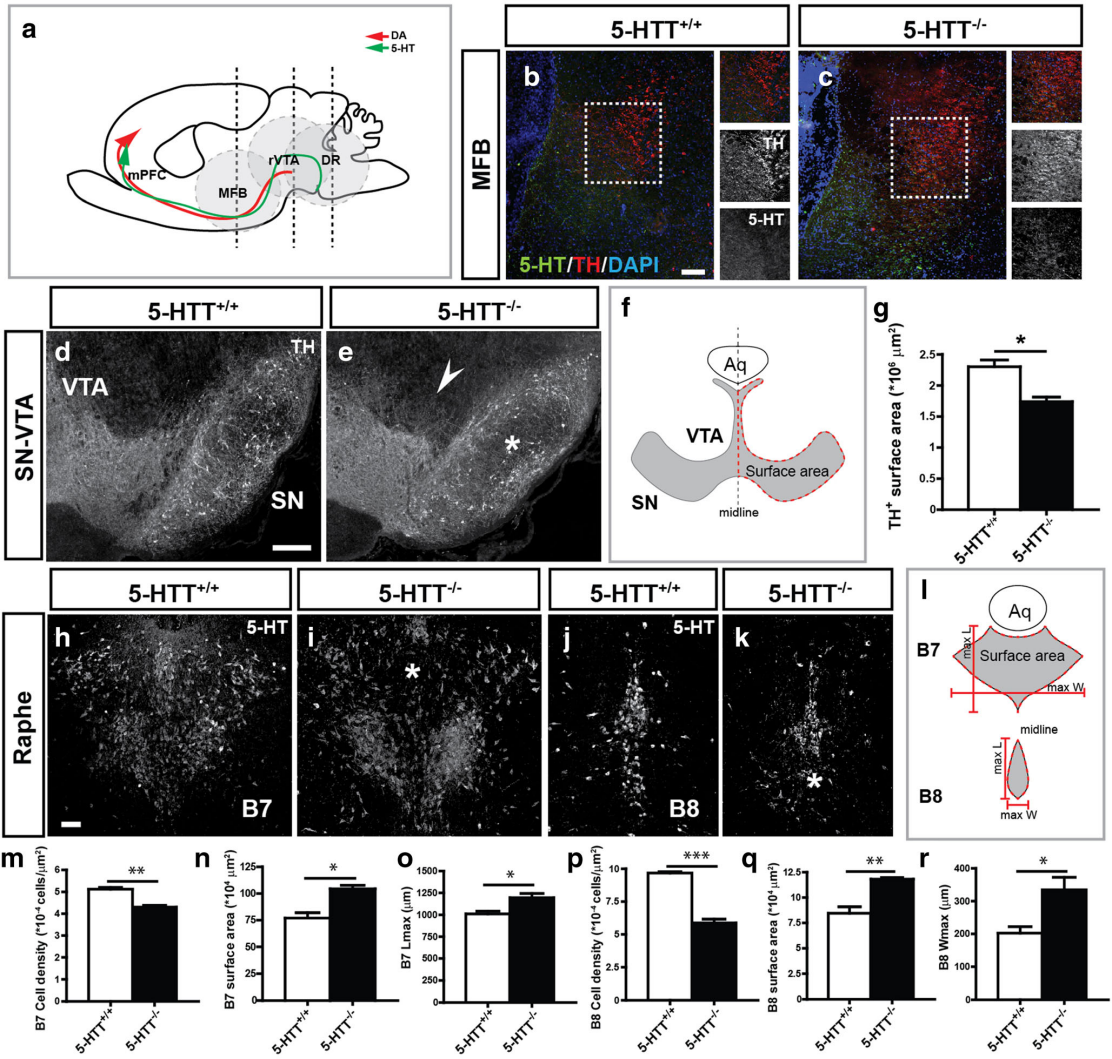
Catecholaminergic system is affected when 5-HT levels are perturbed during development. **a** Schematic representation of a sagittal view of a developing postnatal rodent brain showing the catecholaminergic mesoprefrontal topographic projection (red arrow) and the 5-HTergic rhomben prefrontal topographic projection (green arrow) towards the mPFC highlighting the DA origin rVTA, the 5-HT origin DR, and the MFB (within the gray circles). Enlargements of coronal cryosections of P6 rat brain immunostained for 5-HT (red) and TH (green) and stained with DAPI (blue) showing the MFB of 5-HTT^−/−^ (**c**) compared to 5-HTT^+/+^ animals (**b**) showing a higher level of defasciculation of TH^+^ fibers and a lower level of 5-HT fibers in the 5-HTT^−/−^ pups. Boxed area shows the individual fascicles of the catecholaminergic (middle box) and 5-HT (lower box) system. Enlargements of coronal cryosections of P6 rat brain immunostained for TH (white) showing the rVTA of 5-HTT^−/−^ (**e**) compared to 5-HTT^+/+^ (**d**) animals showing more catecholaminergic fibers exiting the VTA area (arrowhead) and fewer and less organized TH^+^ neurons in the SN (asterisk) of 5-HTT^−/−^pups. **f** Schematic representation of a coronal view of a developing DA midbrain including the VTA and SN showing the measured surface area (surrounded by red dotted lines). Aq aqueduct. (**g**) Graph showing the surface area occupied by TH^+^ fibers/neurons ± SEM which is significantly smaller for 5-HTT^−/−^pups. One-way ANOVA, ^*^p < 0.05. Enlargements of coronal cryosections of P6 rat brain immunostained for 5-HT (white) showing the DR B7 (**h**, **i**) and B8 (**j**, **k**) cell cluster of 5-HTT^−/−^ (**i**, **k**) compared to 5-HTT^+/+^ (**h**, **j**) animals showing irregularities in both cell clusters (asterisks) of 5-HTT^−/−^ pups. **l** Schematic representation of a coronal view of the developing 5-HT raphe area including the B7 and B8 cell clusters showing the measured surface area (surrounded by red dotted lines) and the maximal width (max W) and the maximal lengths (max L) of both clusters (red lines). Aq aqueduct. Quantification of the number of the 5-HT-positive neurons in the B7 (**m**) and B8 (**p**) cluster, the measured surface area of the B7 (**n**) and the B8 (**q**) area, the maximal length of the B7 cluster (**o**), and the maximal width of the B8 cluster (**r**). Bar in **b** and **c**, 200 μm; **d** and **e**, 300 μm; **h**–**k**, 100 μm

As the TH^+^ and 5-HT innervation of the mPFC was affected in 5-HTT^−/−^ animals, the question remained whether also the source of the TH^+^ (rostral VTA or rVTA) and 5-HT^+^ (DR and MnR) prefrontal fibers were affected by changes in 5-HT levels during development. To this end, we measured the surface area of the DA midbrain including the rVTA and the SN of both 5-HTT^−/−^ P6 animals (*n* = 3) and their control counterparts (*n* = 3). The total amount of surface area comprising TH^+^ neurons was significantly lower in 5-HTT^−/−^ animals as compared to controls (*p* = 0.013; Fig. 4g). In addition, there seemed to be more axons leaving the VTA area and TH-positive neurons looked more sparse and disorganized, especially within the SN (Fig. 4d, e). We furthermore measured the total surface area of the raphe B7 and the B8 cluster in P6 5-HTT^−/−^ animals and wildtypes, counted the 5-HT-positive cells in both clusters, and calculated the cell density. The total surface area comprising 5-HT neurons was significantly larger in both the B7 and the B8 cell cluster in 5-HTT^−/−^ animals compared to controls (*p* = 0.01 and *p* = 0.006, respectively) Fig. 4n, q). This was reflected by an increase in length of the B7 cluster and an increase in width of the B8 cluster (*p* = 0.031) and (*p* = 0.037) respectively; Fig. 4o, r). Additionally, the total amount of 5-HT-positive cells within each cluster was significantly lower, resulting in a lower cell density for both cell clusters (*p* = 0.002 and *p* = 0.0002, respectively; Fig. 4m, p).

Together, we can conclude that when 5-HTT is absent during development, the mesoprefrontal catecholaminergic system and rhomben prefrontal 5-HT system are affected.

### Reelin in Relation to 5-HT and Catecholaminergic Signaling

The 5-HT fibers within the MZ have been shown to contact CR cells, and thereby control reelin release [6]. By combining immunostaining for 5-HT and for TH, we observed that there are numerous TH^+^ and 5-HT^+^ projections running through the MZ where the CR reside. The question remains, however, to what extent the absence of 5-HTT interferes with reelin release, either directly or indirectly, through altered 5-HT or other projections.

**Fig. 5:**
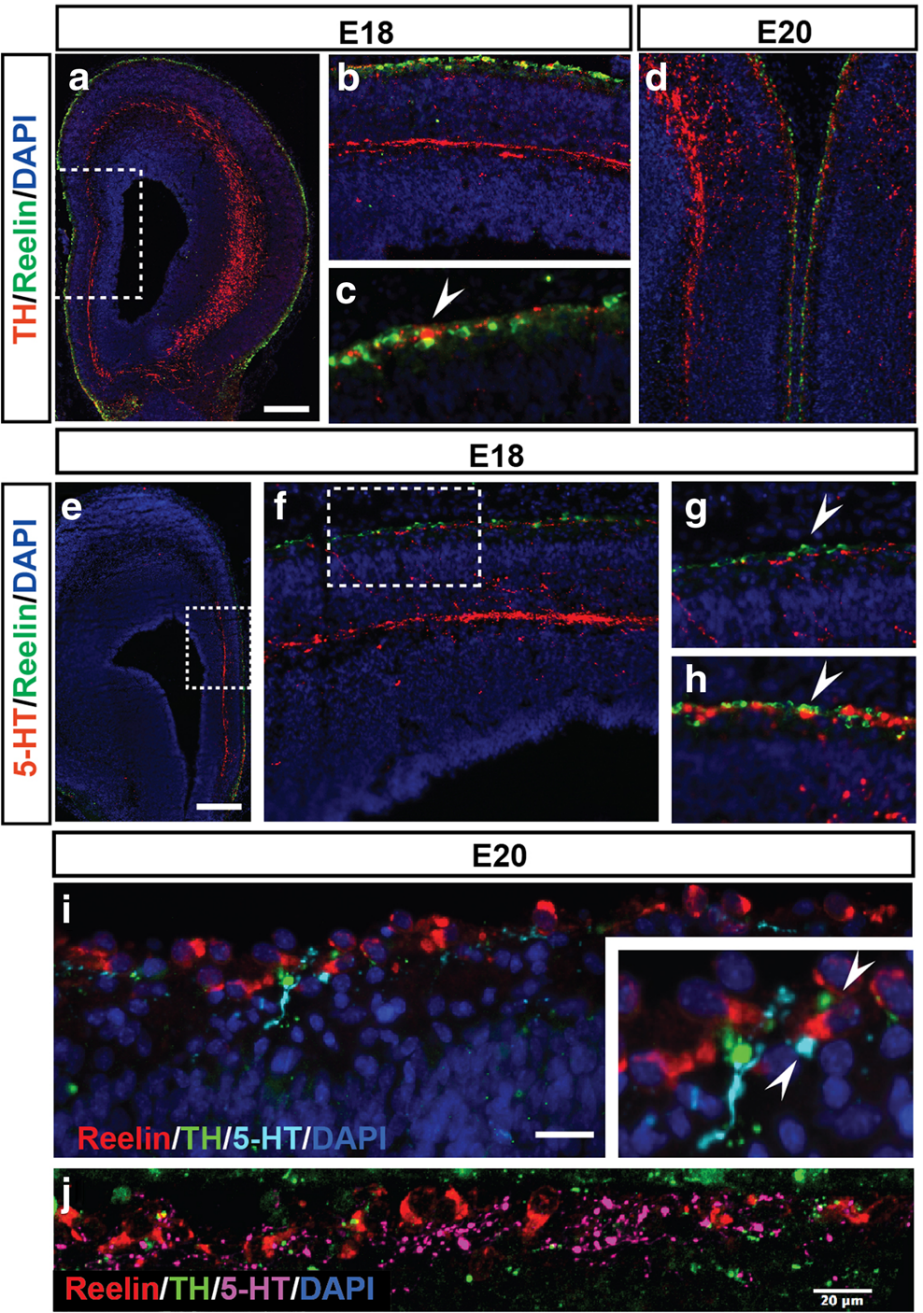
The 5-HT and catecholaminergic systems are in close proximity of the CR cells. Coronal cryosections of E18 (**a**–**c**, **e**, and **F**) and E20 (**d**, **i**) rat brains immunostained for TH (red, **a**–**d**) or 5-HT (red, **e**–**h**) and reelin (green), counterstained with DAPI (blue). **b**, **c** Enlargements of the boxed area in **a** showing the TH fibers in close proximity to the reelin-positive CR cells. **d** Overview of the relation of TH innervation within the mPFC at E20. Coronal cryosections of an E18 rat brain immunostained for 5-HT (red, **e**–**h**, **i**) and reelin (green), counterstained with DAPI (blue). **f** Enlargement of the boxed area in **e** showing the 5-HT fibers in close proximity to the reelin-positive Cajal-Retzius (CR) cells. **g** Enlargement of the boxed area in **f** showing the 5-HT fibers in close proximity to the reelin-positive CR cells. **h** Enlargement of the septal region in **e** showing the 5-HT fibers in close proximity to the reelin-positive CR cells. **i** E20 cryosection stained for TH (green), 5-HT (light blue) and reelin (red) and counterstained with DAPI (blue). Inset shows the close proximity of the TH and 5-HT fibers with the CR cells. **j** Confocal image of the marginal zone showing TH (green) and 5-HT fibers (purple) in close proximity to reelin-positive CR cells (red). Bar in **a** and **e**, 300 μm; **i**, 30 μm; **j**, 20 μm

To visualize the proximity of TH^+^ and 5-HT^+^ fibers with CR cells, we immunostained cryosections of E18 and E20 with either 5-HT or TH in combination with reelin. Throughout cortical regions, including all subdomains of the mPFC, 5-HT- and TH-positive fibers running through the MZ are in close proximity with CR cells (Fig. 5a–h). Confocal images showed that some varicosities were indeed contacting the CR cells (Fig. 5j and Supplemental Figure 2). Triple-labeling the cryosections with TH, 5-HT, and reelin revealed that the 5-HT and TH fibers both contact the CR cells, albeit at different sites (Fig. 5i), suggesting that besides the known 5-HT, also the TH fibers are in the vicinity to be able to influence CR cell output and possible reelin release.

### Prefrontal Cytoarchitecture is Affected in the Absence of the 5-HTT

The fact throughout development, 5-HT and TH fibers reside in close proximity with CR cells and that they are affected in the absence of 5-HTT, made us speculate whether these innervation alterations could have impacted cortical build-up.

**Fig. 6:**
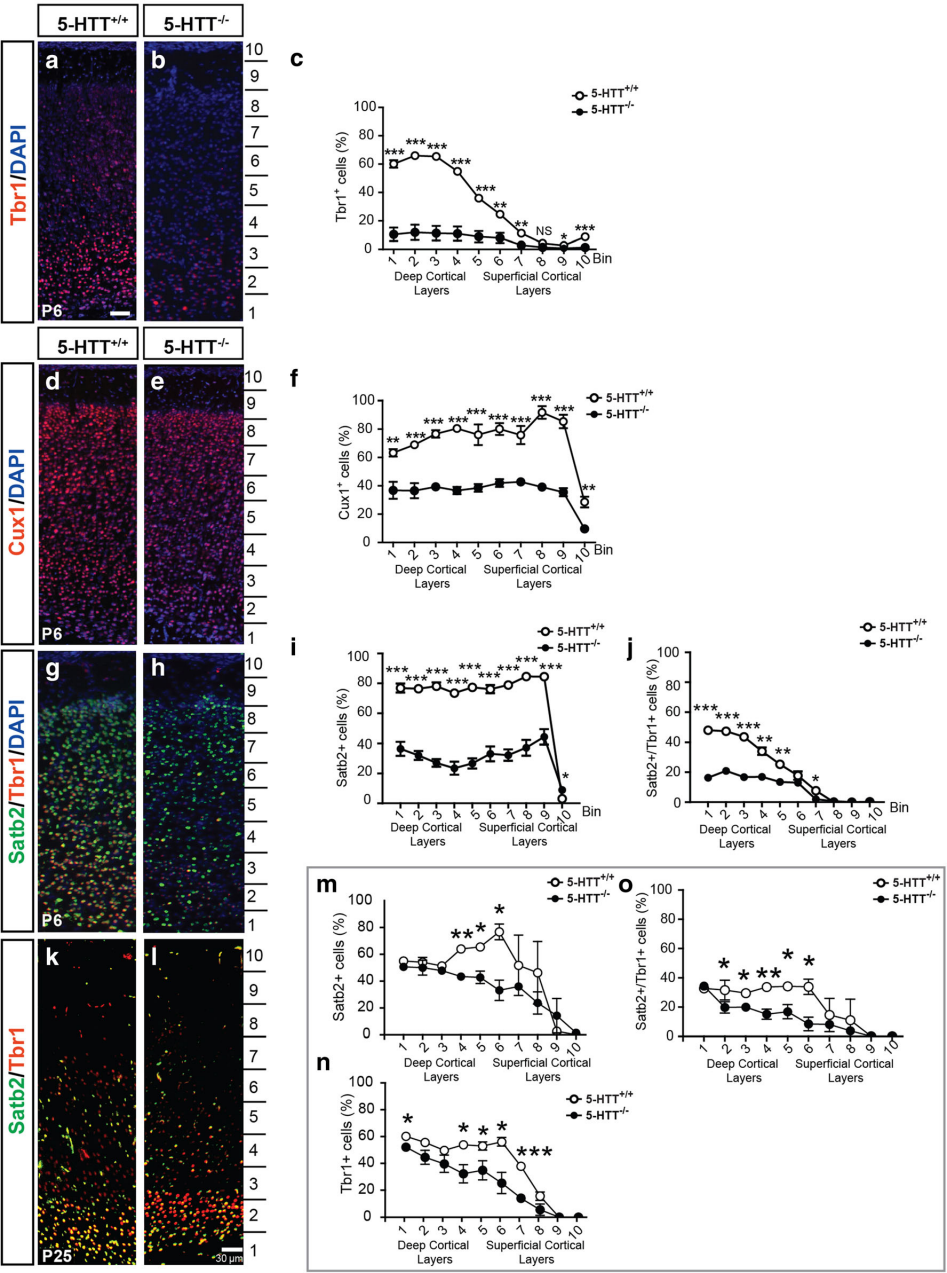
Prefrontal cytoarchitecture is affected in the absence of 5-HTT. Enlargements of cryosections of P6 5-HTT^+/+^ and 5-HTT^−/−^ rat brains showing prefrontal swatches of the PL immunostained for Tbr1 (red, **a**, **b**) or Cux1 (red, **d**, **e**) and counterstained with DAPI (blue). **b**, **e** Quantification of the percentage of Tbr1-positive (**c**) or Cux1-positive (**f**) neurons within the bins indicated in **a**, **b**, **d**, and **e**. **g**, **h** Enlargements of cryosections of P6 5-HTT^+/+^ and 5-HTT^−/−^ rat brains showing prefrontal swatches of the PL double-immunostained for Tbr1 (red) and Satb2 (green) and counterstained with DAPI (blue). **i** Quantification of the percentage of Satb2-positive neurons within the bins indicated in **h**. **j** Quantification of the percentage of Satb2/Tbr1 double-positive neurons within the bins indicated in **h**. **k**, **l** Enlargements of cryosections of P25 5-HTT^+/+^ and 5-HTT^−/−^ rat brains showing prefrontal swatches of the PL double-immunostained for Tbr1 (red) and Satb2 (green). **m** Quantification of the percentage of Satb2-positive neurons within the bins indicated in **l**. **n** Quantification of the percentage of Satb2-positive neurons within the bins indicated in **l**. **o** Quantification of the percentage of Satb2/Tbr1 double-positive neurons within the bins indicated in **l**. Graphs in **c**–**o** show average percentage ± SEM. One-way ANOVA, ^**^p < 0.01, ^***^p < 0.001. Bar in **a**–**l**, 100 μm

To address the question to what extent layer-specific markers were affected, we immunostained mutant and wildtype P6 cryosections for a deep-layer marker, Tbr1 (Fig. 6a–c) and an upper-layer marker, Cux1 (Fig. 6d–f). Indeed, both markers were severely affected in their expression pattern (Fig. 6). The percentage of cells affected was significant in all ten bins, but was most striking for the bins in deeper layers, specifically for Tbr1 (Fig. 6a–c). The total number of Cux1- and Tbr1-positive cells was significantly affected as well (Supplemental Figure 3). This is in coherence with our previous findings demonstrating that Satb2, a layer 2–5 marker was severely affected [10]. The total number of cells (DAPI-positive) was not significantly different in IL (*p* = 0.2), PL (*p* = 0.9), and Cg (p = 0.9); (Supplemental Figure 3), suggesting that the altered layer marker expression is due to an altered identity.

To investigate marker expression at a later developmental age, we immunostained mutant and wildtype P25 cryosections for two different deep-layer markers; Tbr1 and Satb2 (Fig. 6k–o). Expression levels of both markers were more restricted to the deeper layers in the wildtype brains and most affected when 5-HTT was absent (Fig. 6m–o). This suggests that, even though the percentage of cells expressing deep-layer markers catch up a bit at P25 reflecting some sort of developmental delay, a large portion will not be able to express these markers.

**Fig. 7:**
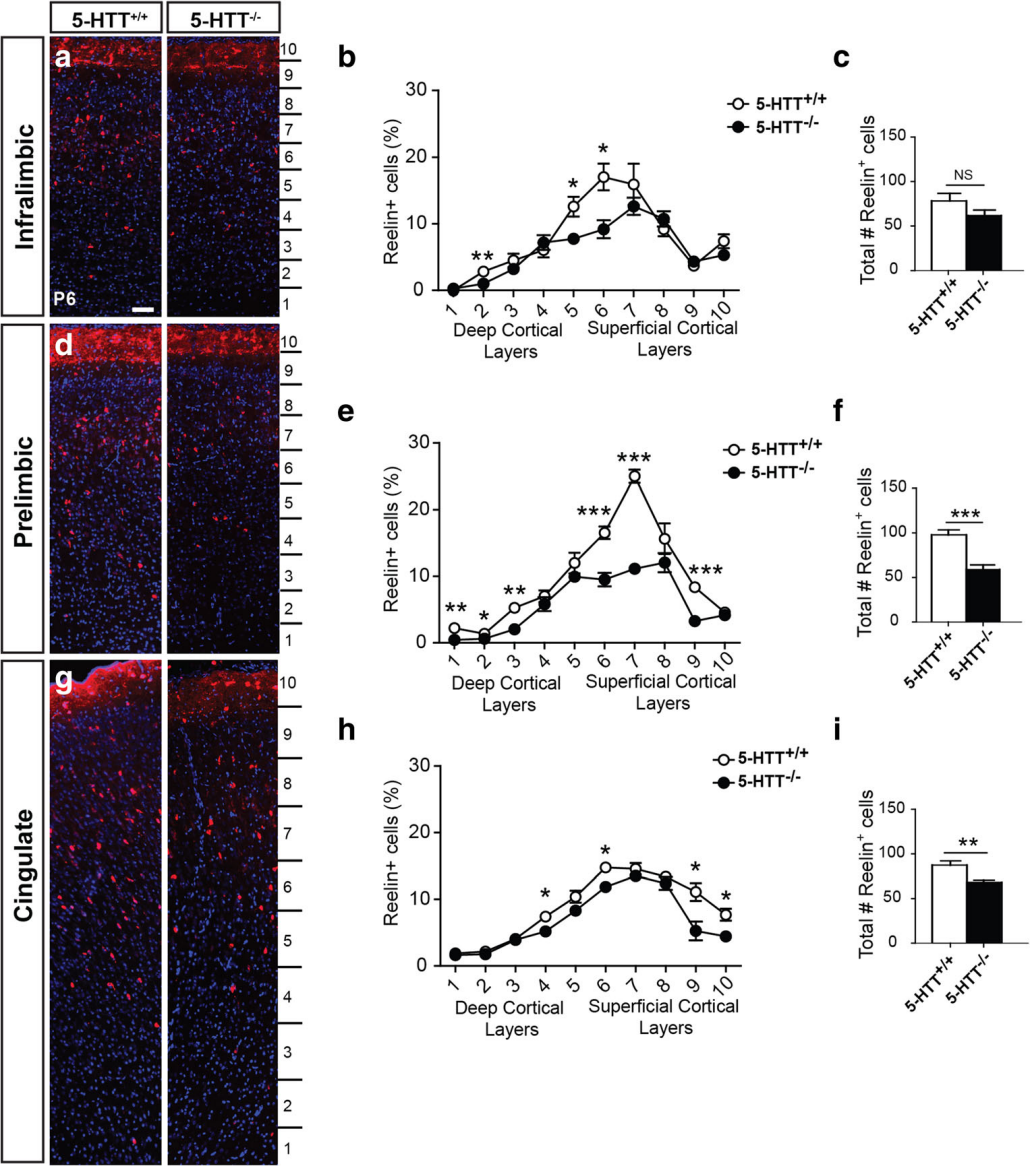
The number of reelin-positive cells is diminished in the absence of 5-HTT. Enlargements of cryosections of P6 5-HTT^+/+^ and 5-HTT^−/−^ rat brains showing prefrontal swatches of the IL (**a**), PL (**d**), and the Cg (**g**) immunostained for reelin (red) and counterstained with DAPI (blue). Quantification of the percentage of reelin-positive neurons within the bins indicated in **a**, **d**, and **g** in the IL (**b**), PL (**e**), and Cg (**h**) of 5-HTT^−/−^ compared to 5-HTT^+/+^ pups confirming the qualitative observations. Graphs in **b**, **e**, and **h** show average percentage of reelin-positive neurons normalized to total number of cells per bin ± SEM. One-way ANOVA, ^*^p < 0.05, ^**^p < 0.01, ^***^p < 0.001. Quantification of the total number of reelin-positive cells over the complete length of the prefrontal swatch in the IL (**c**), PL (**f**), and Cg (**i**) of 5-HTT^−/−^ (black bar) compared to 5-HTT^+/+^ pups (white bar). Graphs in **c**, **f**, and **i** show average number ± SEM. One-way ANOVA, ^**^p < 0.01, ^***^p < 0.001. Bar in **a**–**g**, 100 μm

Reelin-positive interneurons will dispense throughout the cerebral wall in early postnatal ages [36, 37, 63]. Even though the ontogeny of reelin-positive CR cells and the reelin-positive GABAergic interneurons differ in their ontogeny [64], we pursued to find out whether the number and integration of reelin-positive cells was affected by the lack of 5-HTT during the course of development. We counted the reelin-positive cells in the P6 mutant and the control animals. We discovered that in all subdomains of the mPFC, the number and distribution of reelin-positive neurons was affected in the 5-HTT^−/−^ animals as compared to wildtypes (Fig. 7). In the IL, only bins 2, 5, and 6 showed lower number of reelin-positive cells (Fig. 7a, b), while in the PL, all bins except for 4, 5, and 8 showed significant lower numbers of reelin-positive cells (Fig. 7d, e). Within the Cg, bins 4, 6, 9, and 10 showed significant lower numbers of reelin-positive cells (Fig. 7g, h). Together, we found a decrease in the total number of reelin-positive cells in all subdomains although the number did not reach significance within the IL (Fig. 7c, f, and i). This suggests that the integration and/or number of reelin-positive interneurons within the subdomains of the mPFC is affected as well.

**Fig. 8:**
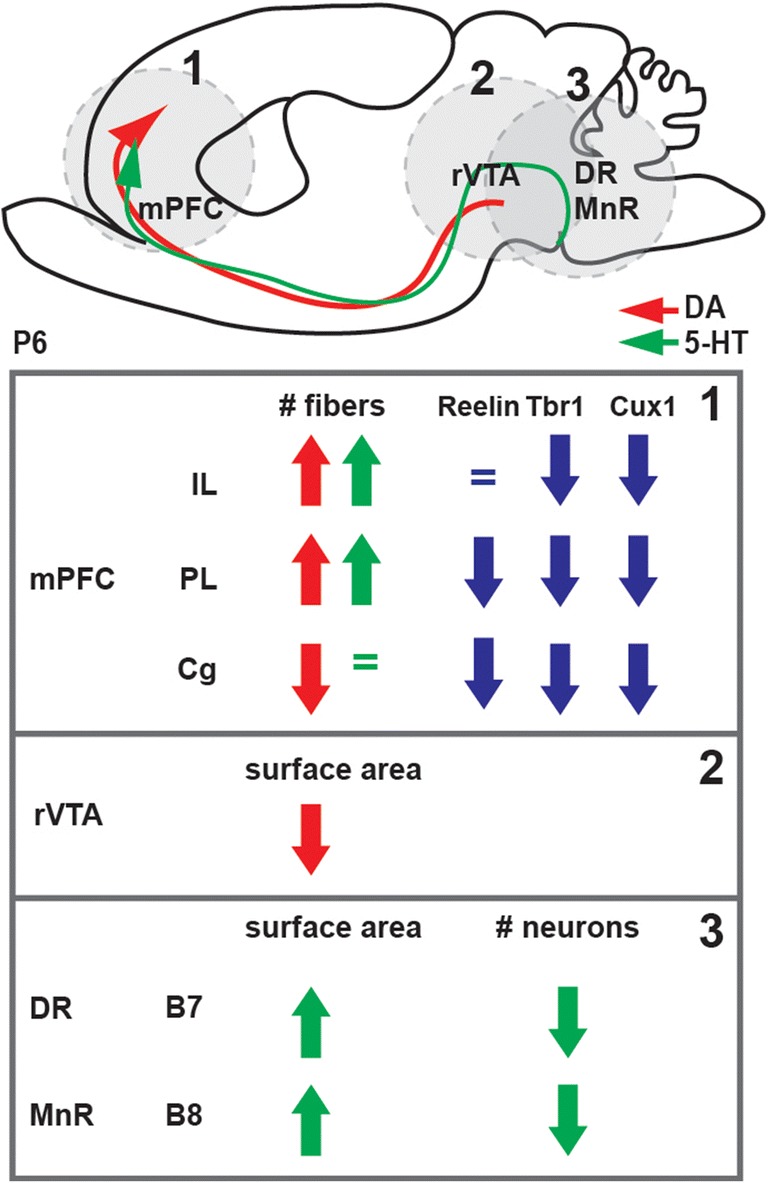
Schematic overview of the results observed in the 5-HTT rat model. DA dopaminergic, Cg cingulate, DR dorsal raphe, 5-HT serotonergic, IL infralimbic, MnR medial raphe; mPFC medial prefrontal cortex, PL prelimbic, rVTA rostral ventral tegmental area

In all, we observed a strong interdependence between the developing 5-HT and catecholaminergic system. In the absence of 5-HTT, we found significant differences in the shape (rVTA) and in the shape and content (DR/MnR) of the origins of both neural systems, a striking increase of both 5-HT and catecholaminergic innervations of the mPFC, altered deep-layer identity of mPFC neurons, and a decrease in reelin-positive cells, and which is summarized in Fig. 8.

## Discussion

In this study, we show the coinciding development of the 5-HT and the catecholaminergic systems between E16 and P25 within their origin, their outgrowing projections through the MFB and their common projection target, the mPFC. In addition, our results demonstrate that in the absence of the 5-HTT and next to the 5-HT, also the catecholaminergic system and their projections towards the mPFC are altered. We further demonstrate that within the mPFC, the TH and 5-HT fibers are in close proximity to reelin-containing CR cells, and are different in number when 5-HTT is lacking. We also observe that, with differences in 5-HT/TH innervation of the mPFC, prefrontal cell identity is altered. Altogether, these data suggest that there is a functional interplay between the 5-HT and catecholaminergic systems during development with an effect on the proper cytoarchitecture of the PFC.

### Catecholaminergic and 5-HT Control of Prefrontal Development

It is now well accepted that 5-HT plays an important role during neurodevelopment and that any disturbance of the system could add to the risk of developing neuropsychiatric conditions [30, 65–67]. There is considerable genetic diversity among 5-HT neurons resulting in a vast and meticulously constructed network projecting to a large variety of targets [8, 24, 68, 69]. The development of this heterogeneous pool of 5-HT neurons is under the control of intrinsic factors (e.g., transcription factors) and in interplay with extrinsic factors (e.g., guidance cues or cell adhesion molecules) that can steer the targeting projections [70–75]. Prefrontal 5-HT neurons arise in the rostral raphe cluster and the ascending axonal projections bundle up within the MFB and the fascicles traverse through the septal area towards the mPFC [6, 23, 76]. Here, they are bundled in two paths; one within the superficial MZ and one on top of the SP underneath the CP which they innervate after a short waiting period [6, 23, 77]. Essentially, the same developmental trajectory holds true for the mesoprefrontal catecholaminergic projections, although the catecholaminergic system reaches the mPFC earlier [42, 43, 49]. Within the MZ and presumptive layer I of the mPFC, we observed 5-HT- and TH-positive varicosities in very close proximity of the reelin-positive CR cells. Synaptic structures, stained with either pre- or postsynaptic markers, could shed light on to what degree the CR cells receive TH- and/or 5-HT-synaptic inputs during development. In addition, varicosities do not necessarily have to imply synapses but can be a reflection of the complex that uses diffuse/volume transmission to communicate [78–80]. Nonetheless, there is certainly a spatial closeness of 5-HT^+^ as well as TH^+^ fibers that could imply the ability to influence CR output and maybe even reelin release. Reelin is known for its ability to direct cortical layer formation [81, 82]. However, the developmental role of the effect of 5-HT, DA, or other neurotransmitters on reelin release needs to be further characterized.

### Direct or Indirect Effects of 5-HTT on Cortical Integrity

One critical way by which extracellular 5-HT levels can be controlled is through expression of 5-HTT. The transporter can clear 5-HT from the synaptic cleft to maintain homeostasis [83]. Remarkably, the expression of 5-HTT is already quite robust in early development, even before serotonergic axons have reached their targets [60, 84]. What does this imply? It is known that there is an extra-embryonic source of 5-HT from the placenta that could regulate certain aspects of central nervous system development [30, 85, 86]. Yet, many questions remain. Narboux-Nème and colleagues [60] elegantly showed a transient 5-HTT expression within layers II, V, and VI of the mPFC at E15.5, thus even before 5-HT fibers reach the mPFC. Could it be that these deep-layer cortical neurons are most affected by the absence of 5-HTT and the resulting elevation of extracellular 5-HT? We indeed observed that in both layers V and VI as well as more superficial aspects of the prefrontal subareas, 5-HT- and TH-innervation were altered in the absence of 5-HTT. In addition, in our experiments, the number of predominantly deep-layer but also of the superficial-layer neurons was affected in the absence of 5-HTT at P6. At P25, however, we still see this effect for two different deep-layer markers (Tbr1 and Satb2); however, there is some percentage expression that does express these markers in the mutant suggesting a developmental delay. There are no indications that proliferation or migration (data not shown) was affected. Altamura and colleagues showed that in 5-HTT-deficient mice there are differences in cell density and layer thickness [87]. Does this mean that there is a different window of expression of layer-specific markers Cux1 and Tbr1 when 5-HTT is absent? At this point, it is hard to conclude whether this change in expression of layer markers is due to cell-autonomous effects (absence of 5-HTT) or cell non-autonomous effects (differences in innervation patterns/reelin effect), or a combination of both. Experiments using conditional mutants [88] that have a cell-type-specific deletion of 5-HTT could shed more light on this.

### Interaction of the Developing Catecholaminergic and 5-HT Systems

Numerous studies have proven that there is an interdependence between 5-HT and DA [11, 15, 16, 18, 89], although less is known about this phenomenon during development. Both systems are able to influence neurodevelopmental events such as proliferation, migration, and differentiation [30, 51, 62, 90]. But can one system be facilitated by the other during development? We showed that the TH^+^ projections within subdomains of the mPFC are altered in absence of the 5-HTT. Expression of 5-HTT has been found within virtually all DR neurons [60, 84, 91]. Are 5-HTT-deficient DR neurons able to alter the course of TH^+^ projections? And if yes, what are the exact neurodevelopmental events of the developing DA neurons that can be influenced by the 5-HT system and at what developmental time points? Alongside the changes in 5-HT receptor expression [92–99], the expression of DA receptors and transporters might be altered as a consequence of a disrupted 5-HT system and DA system. This can result in changes in system sensitivity and excitability which would have extreme consequences for the maturation of cortical cells. For example, reelin-positive interneurons express the 5-HT3A receptor [64, 100]. Could changes in the 5-HT projection system have led to the changes we observed in the number and distribution of the reelin-positive cells? We need to have a complete picture of the spatial and temporal aspects of these developing systems in order to be able to design preventive measures or curative treatment without any side effects.

### Guidance of Interconnected Systems

The 5-HT projections to target-selective forebrain regions are under the control of classical guidance molecules [25, 70, 75]. The 5-HT system is furthermore capable of modulating the responsiveness of axons to guidance cues such as netrins [62]. However, the catecholaminergic system reaches the mutual forebrain targets earlier than the 5-HT system does. Can it be that the altered catecholaminergic system development due to the absence of 5-HTT influences the developing mPFC earlier than the 5-HT and in a different manner? Or is it the absence of 5-HTT in target areas and within guidepost positions along the way that has changed guidance cue expression or the responsiveness of the TH^+^ fibers? Even though the DR and MnR project to different targets, their development involves a common guidance family [101]. The differential expression of EphA5 and ephrina5, and consequential difference in Eph-ephrin signaling, steers the region-specific 5-HT innervation. Whether developing catecholaminergic projections within the same temporal and spatial window are also responsive to the same guidance cues as the 5-HT axons headed towards the mPFC remains to be established.

Neurodevelopmental processes may diverge in different brain regions and at various developmental time points. For example, the ontogeny of neurotransmitter systems can be affected by risk factors and aberrant projections may result [1, 9, 10, 22, 50]. Depending on the type of risk factor involved and their sensitive windows, which control the timing of when a disorder becomes overt, it is either the mPFC itself or its connected brain areas that may maldevelop. Yet, the net effect of faulty projections on other developing systems remains to be determined. Longitudinal studies at the systems level, including a complete inventory of the expression of a variety of neurotransmitter receptors and transporters in relation to the developing projections traveling together and their actions within the mPFC are needed. This would generate an important wealth of knowledge in order to understand the complexity of these interacting systems during development.

## Conclusions

Our study shows a functional interplay between the 5-HT and catecholaminergic systems during development. As expected, due to the absence of the 5-HTT the 5-HT system was disturbed but we found that the catecholaminergic system was perturbed as well, together resulting in an altered maturation of the mPFC. Overall, the striking observation of both 5-HT and catecholaminergic hyperinnervation of prefrontal subregions highlights the need for precise system-oriented dissection of neural systems that concomitantly develop. The removal of only one building block may destabilize a plethora of interacting neurodevelopmental systems leading to impairment of cognitive functioning. This calls for more studies on the dissection of neurotransmitter systems-specific consequences on adult behavior to eventually allow the design of better treatment strategies for neuropsychiatric disorders.

## Electronic Supplementary Material

Supplemental Figure 1 5-HT and catecholaminergic system is affected when 5-HT levels are perturbed during development. (A, B, C) Quantification of the 5-HT fiber length (in μm) within the bins indicated in A, D and G of Fig. 1 in the IL (A), PL (B) and Cg (C) of 5-HTT^−/−^ compared to 5-HTT^+/+^ pups. Graphs in A, B and C show average total length of 5-HT-positive fibers per bin ± SEM. One-Way ANOVA, ^*^p < 0.05, ^**^p < 0.01, ^***^p < 0.001. (D) Schematic representation of the mPFC with enlargement of the binning of the cortical swatch (Cg as an example) in order to perform the quantifications. (E) Enlargement of Fig. 4D and E of the arrowhead-indicated area within the rVTA (also schematically shown in the left upper corner) showing more aberrant fibers in the 5-HTT^−/−^ animals. (PNG 1.60 mb)

Supplemental Figure 2 Close proximity of the 5-HT and catecholaminergic system with CR cells. Confocal images showing 5-HT (purple) and TH (green) in close proximity with reelin-positive (red) CR cells. (PNG 815 kb)

Supplemental Figure 3 Prefrontal cytoarchitecture is affected in absence of 5-HTT. (A, B, C) Quantification of the total number of Cutl1- (A), Tbr1- (B) and DAPI-positive cells of in the IL, PL and Cg of 5-HTT^−/−^ compared to 5-HTT^+/+^ pups. One-Way ANOVA, ^***^p < 0.001. (PNG 382 kb)
