# Supplementary figures and images for: Perturbed Developmental Serotonin Signaling Affects Prefrontal Catecholaminergic Innervation and Cortical Integrity
Source: Mol Neurobiol. 2018 Jun 9;56(2):1405–20. doi: 10.1007/s12035-018-1105-x (PMC6400880; doi:10.1007/s12035-018-1105-x)

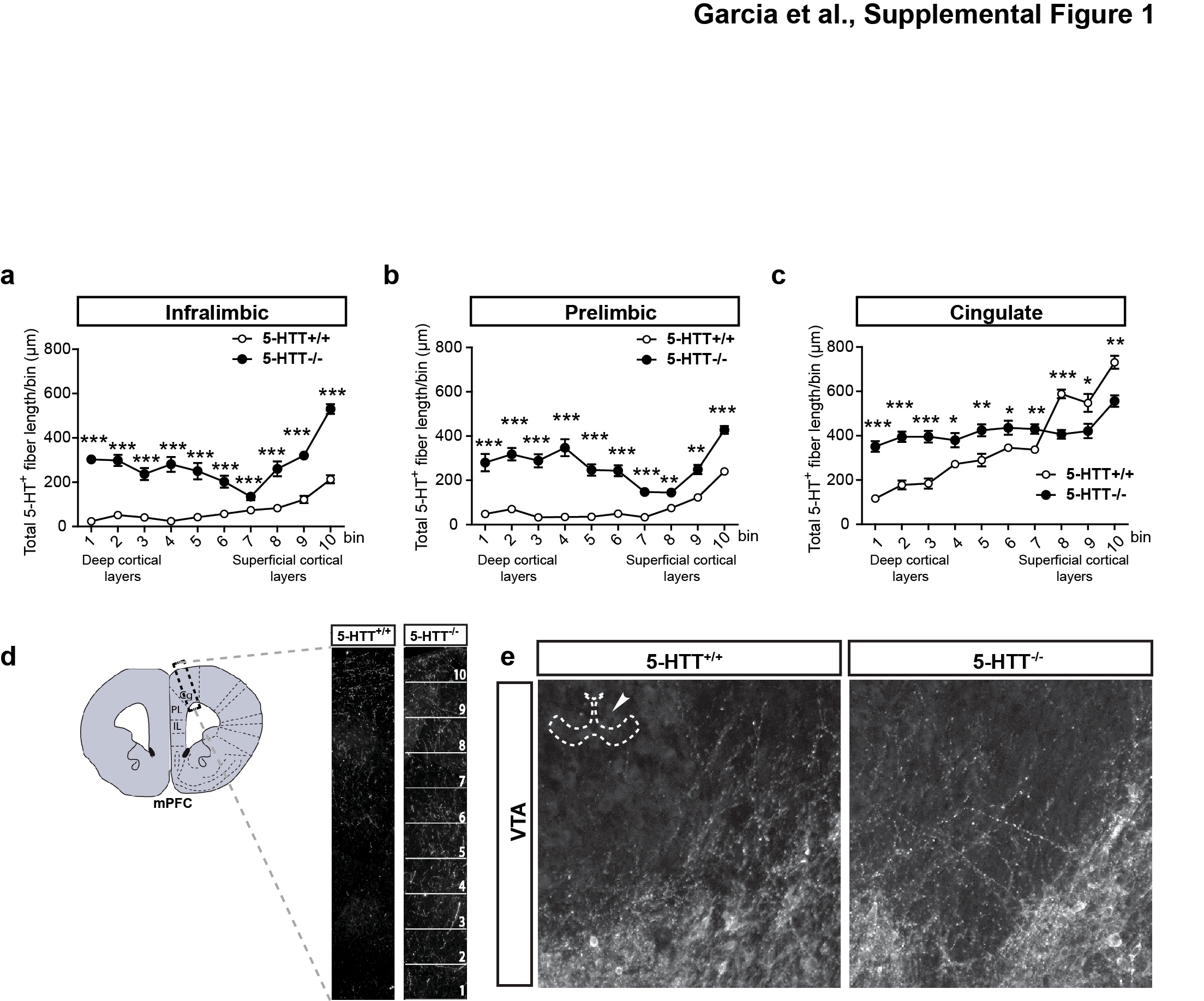

Supplement: Supplementary file 1 — 5-HT and catecholaminergic system is affected when 5-HT levels are perturbed during development. (A, B, C) Quantification of the 5-HT fiber length (in μm) within the bins indicated in A, D and G of Fig. 1 in the IL (A), PL (B) and Cg (C) of 5-HTT−/− compared to 5-HTT+/+ pups. Graphs in A, B and C show average total length of 5-HT-positive fibers per bin ± SEM. One-Way ANOVA, *p < 0.05, **p < 0.01, ***p < 0.001. (D) Schematic representation of the mPFC with enlargement of the binning of the cortical swatch (Cg as an example) in order to perform the quantifications. (E) Enlargement of Fig. 4D and E of the arrowhead-indicated area within the rVTA (also schematically shown in the left upper corner) showing more aberrant fibers in the 5-HTT−/− animals. (PNG 1.60 mb) [file 12035_2018_1105_MOESM1_ESM.png]

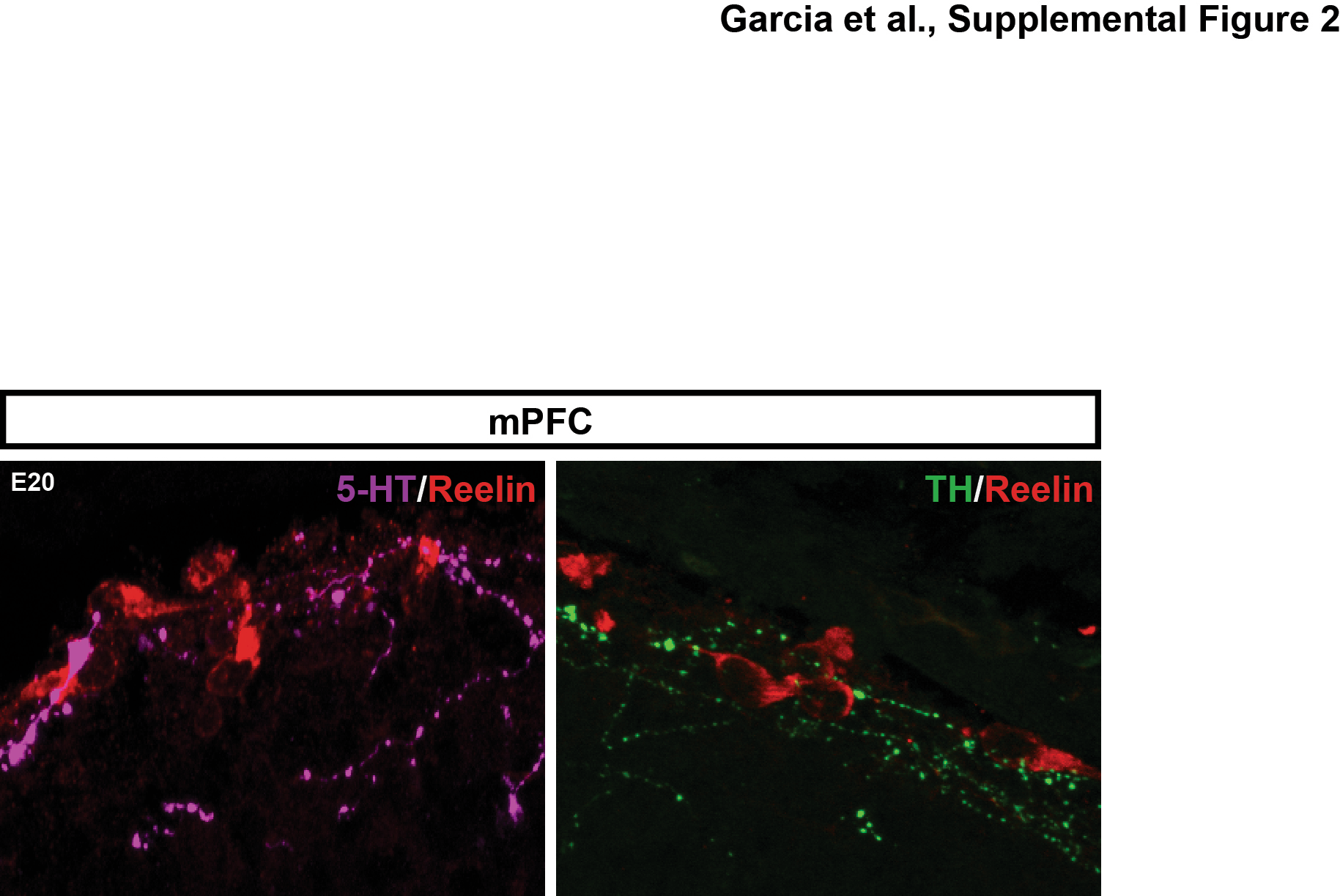

Supplement: Supplementary file 2 — Close proximity of the 5-HT and catecholaminergic system with CR cells. Confocal images showing 5-HT (purple) and TH (green) in close proximity with reelin-positive (red) CR cells. (PNG 815 kb) [file 12035_2018_1105_MOESM2_ESM.png]

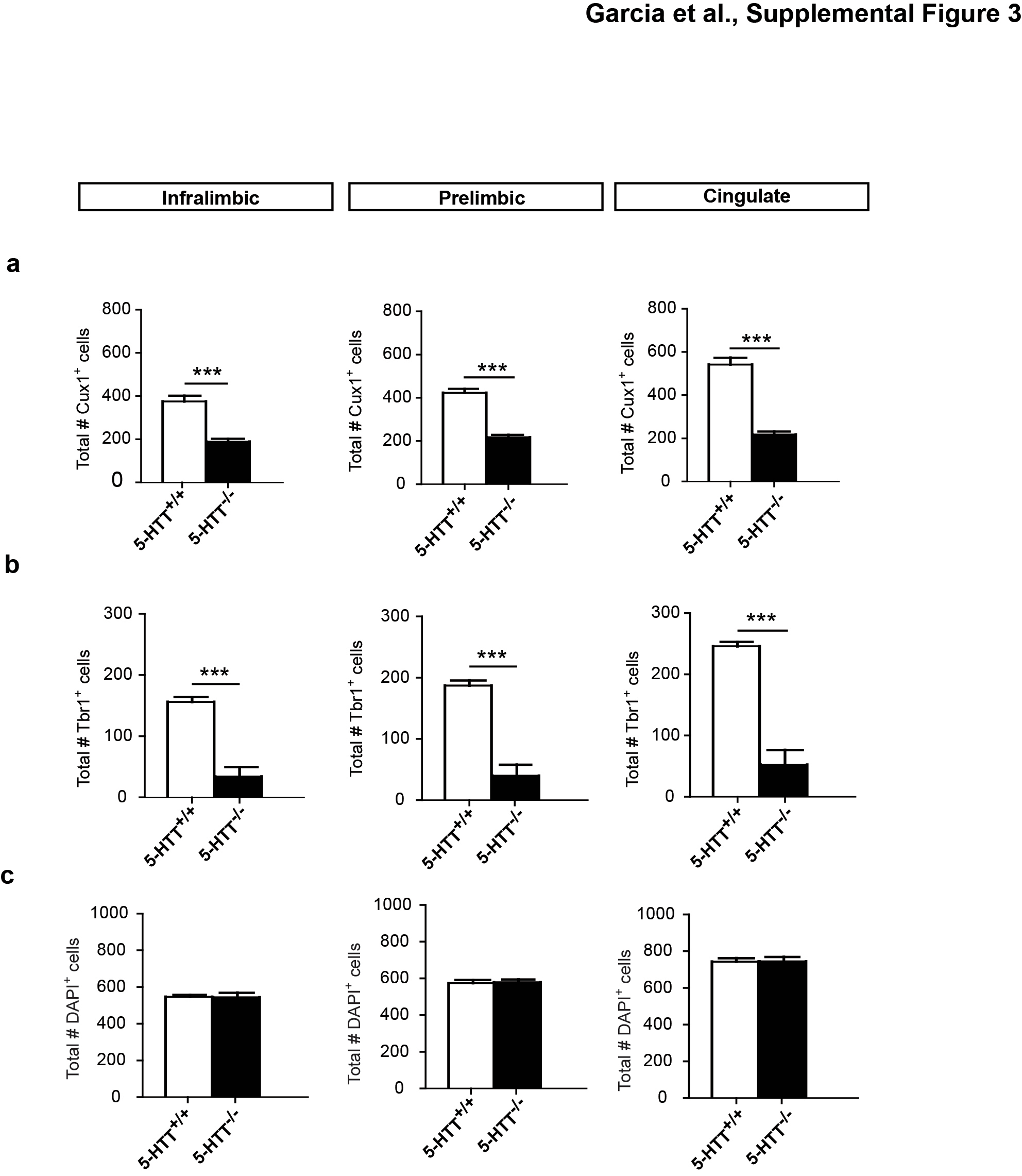

Supplement: Supplementary file 3 — Prefrontal cytoarchitecture is affected in absence of 5-HTT. (A, B, C) Quantification of the total number of Cutl1- (A), Tbr1- (B) and DAPI-positive cells of in the IL, PL and Cg of 5-HTT−/− compared to 5-HTT+/+ pups. One-Way ANOVA, ***p < 0.001. (PNG 382 kb) [file 12035_2018_1105_MOESM3_ESM.png]
